# Inhibitory Receptors and Pathways of Lymphocytes: The Role of PD-1 in Treg Development and Their Involvement in Autoimmunity Onset and Cancer Progression

**DOI:** 10.3389/fimmu.2018.02374

**Published:** 2018-10-17

**Authors:** Elena Gianchecchi, Alessandra Fierabracci

**Affiliations:** ^1^Infectivology and Clinical Trials Research Department, Children's Hospital Bambino Gesù, Rome, Italy; ^2^VisMederi S.r.l., Siena, Italy

**Keywords:** PD-1, Tregs, autoimmunity, T1D, cancer

## Abstract

Regulatory T (Treg) cells represent a subpopulation of suppressor CD4^+^ T cells critically involved in the establishment of peripheral tolerance through the inhibition of effector T (Teff) cells and the suppression of the immune-mediated tissue destruction toward self-antigens. Treg generation, their suppressive properties and also Treg-Teff cell interactions could be modulated at least in part by programmed cell death-1 (PD-1) expression on their surface and through binding between PD-1 and programmed cell death ligand-1 (PD-L1). Defects involving PD-1 and Tregs can lead to the development of pathological conditions, including autoimmune disorders or promote cancer progression by favoring tumor evasion from the host immune response. At the same time, PD-1 and Tregs could represent attractive targets for treatment, as demonstrated by the therapeutic blockade of PD-L1 applied for the management of different cancer conditions in humans. In the present Review, we focus specifically the role of PD-1/PD-L1 on Treg development and activity.

## Introduction

The programmed cell death 1 (PD-1, CD80) molecule is a 55kDa type I transmembrane protein (1) belonging to the immunoglobulin superfamily. PD-1 bears the immunoreceptor tyrosine-based inhibitory motif (ITIM) in its cytoplasmic region (2), which is present also in several immunological negative receptors such as killer cell immunoglobulin-like receptors (KIRs) on natural killer (NK) cells and cluster of differentiation (CD) 22 and FcγRIIB on B cells. PD-1 was isolated for the first time in 1992 by the group of Ishida from a murine T cell hybridoma undergoing programmed cell death (2). Although murine PD-1 (mPD-1) mRNA expression is associated with activation-induced apoptosis in murine T cell hybridomas, PD-1 binding does not lead to cell death, instead it causes cell cycle blockade. Merely 10 years later from PD-1 discovery, the physiological role of this pathway and wherein is involved remain to be elucidated.

In more detail, the study involving PD-1 deficient mice has revealed a key role for PD-1 as a negative regulator of immune responses (3). Murine models with different genetic background showed the development of different autoimmune conditions characterized by delayed onset, organ-specific effects and incomplete penetrance. In particular, PD-1-deficient C57BL/6 mice spontaneously developed lupus like arthritis, splenomegaly, and glomerulonephritis; furthermore, these animals showed an increased number of B and myeloid cells, and enhanced IgA, IgG2b, and IgG3 levels in the serum (4). In Balb/c mice, PD-1 deletion caused a peculiar autoimmune phenotype already at 5 weeks of age, characterized by dilated cardiomyopathy, gastritis, and elevated circulating levels of troponin reactive IgG1 (5). In non-obese diabetic (NOD) mice, PD-1 deficiency accelerated subacute Type I diabetes (T1D) development, but it did not cause the onset of other autoimmune conditions. Hence it promoted the inherent autoimmune susceptibility in this background without modifying its specificity. Finally, lethal myocarditis developed in mice with Murphy Roths Large (MRL) background (6, 7). It is supposed that PD-1 deficiency could promote tissue-specific autoimmunity inherent in the strain by favoring the activation of those T cells that in *Pdcd1*^+/+^ mice were found anergic (8).

The homolog of murine PD-1 (mPD-1) is PD-1 (CD279) in humans, which is characterized by 60% identity with mPD-1 (9–11). PD-1 expression is identified on a limited population of CD4^−^CD8^−^ double negative (DN) thymocytes and is present on several cell types, such as activated T and B lymphocytes (12, 13), NK T cells, regulatory T cells (Tregs) (14), activated monocytes, and dendritic cells (DCs) in both humans and mice (15) and on human germinal center-associated T cells (16). The expression of PD-1 on the surface of activated T cells occurs during the initial activation phase. However, PD-1 regulates the immune response at a later stage during the peripheral tissue infiltration by effector T cells (Teffs). This is different in respect to Cytotoxic T Lymphocyte Antigen-4 (CTLA4) which represents another key immune checkpoint and is mainly involved in the modulation of the magnitude during the initial stages of T cell activation (priming) in the regional lymph node.

Two ligands, programmed cell death ligand-1 (PD-L1) (B7-H1, CD274), and PD-L2 (B7-DC, CD273), are recognized by PD-1, however PD-L1 expression is wider than PD-L2 expression. Among the cells that constitutively express PD-L1 there are T and B lymphocytes, DCs, macrophages, mesenchymal stem cells, bone marrow-derived mast cells (17) and activated Tregs. Furthermore, it has been detected also at sites of immune privilege, such as the eye, placenta, and testes (15). PD-L1 expression has been described also on tumor cells (18). Conversely, the expression of PD-L2 is restricted mainly on macrophages and DCs. The engagement of PD-1 by its ligands provides inhibitory signals involved in the regulation of central and peripheral tolerance through the inhibition of cytokine synthesis (19), T cell proliferation and cytotoxic activity. Mazanet et al. (19) observed that the negative regulation of T lymphocytes activated by endothelial cells (EC) and involving PD-1 signaling pathway did not involve activation markers, but affected selectively the production of cytokines. Furthermore, this inhibitory effect was directly correlated with the strength of the primary stimulus.

During the process of central tolerance, PD-1 and PD-L1 expression has been detected on the surface of maturing thymocytes. The thymus shows a wide expression of PD-L1, whereas PD-L2 expression has been observed on thymic medulla. The finding that thymocyte transition from DN to the CD4^+^CD8^+^ double positive (DP) stage was considerably promoted by PD-1 deficiency has allowed to speculate that PD-1 pathway could modulate the repertoire of mature T lymphocytes; the phenomenon was observed in both T cell receptor (TCR) transgenic lines and in recombination activating gene (RAG)-2^−/−^ mice receiving anti-CD3 mAb. This could occur by negatively modulating the threshold for β selection and regulating the positive selection (20).

The pathway PD-1/PD-L1 has been recognized to modulate and maintain peripheral CD4, including CD4^+^ Tregs and CD8^+^ T cell tolerance at several levels, in particular both T lymphocyte stability and integrity. More specifically, it can down-regulate self-reactive T cells during the presentation of self-antigen by DCs (21, 22). PD-1 is also able to directly promote interleukin-10 (IL-10) secretion by T cells (23) and inhibit the maturation of DCs (24). PD-L1/PD-1 interaction shows a critical role also for the establishment of fetus tolerance (25, 26).

Nishimura et al. (4) first highlighted a correlation between PD-1 pathway and the onset of autoimmunity (4). More specifically, PD-1 disruption resulted in the spontaneous development of lupus-like autoimmune disease associated with glomerulonephritis and predominant IgG3 deposition in aged C57BL/6(B6)-PD-1^−/−^congenic mice. This phenomenon is putatively due to the chronic breakdown of peripheral self-tolerance. Conversely, B6-PD-1^+/+^ mice at the same age did not show arthritis and presented only marginal and probably age-associated glomerular lesions (4).

**Figure 1:**
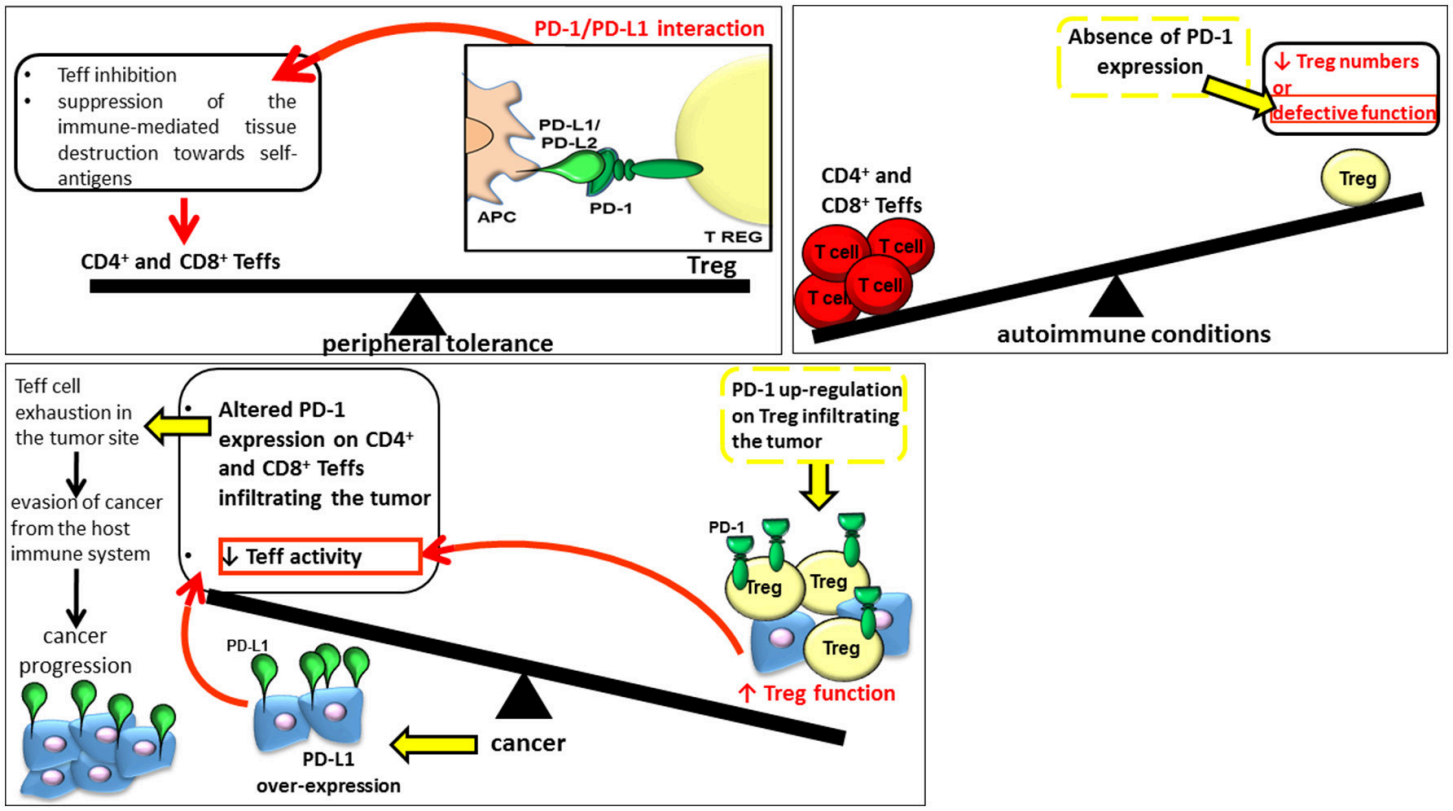
The role of PD-1 and Tregs in peripheral tolerance, onset of autoimmune conditions and cancer progression.

Recent investigations have moreover supported the presence of an association between defects affecting this pathway and the onset and progression of several autoimmune conditions (Figure 1) (27, 28). The PD-1-PD-L1/L2 pathway has a protecting effect for the host toward hyper-activated Teff cells in case of microbial infections inhibiting both Teff proliferation and capacity which could otherwise lead to chronic infection; conversely, in case of cancer, this signaling pathway can favor cancer progression through strong inhibitory mediators (Figure 1) (29, 30). In this respect, PD-1 immuno-checkpoint blockade exerted significant antitumor effects in several malignancies, especially in melanoma patients, attracting much attention in oncotherapy in the last years (31). The pharmacological treatment based on PD-1-PD-L1/PD-L2 immune checkpoint inhibitors (ICIs) is able to restore the number of Teff cells and promote their cytotoxic immune responses directed against chemotherapy-refractory tumors and restore the activity of exhausted CD8^+^ T cells in chronic viral infections (32). The therapy promotes also the synthesis of pro-inflammatory cytokines restoring the ongoing tumor immunity (33–36). On one side, if treatment with anti-PD-1/PDL-1 agents was responsible for better survival in several different cancers, on the other, after such treatments, ICIs can cause the onset of inflammatory side effects affecting any organ system, conditions defined as immune-related adverse events (IrAEs) (37). In addition to organ specific IrAEs, more general AEs related to immune activation, such as fatigue, rash and diarrhea, as well as AEs potentially attributable to systemic inflammation, especially musculoskeletal manifestations, have been reported in patients receiving anti-PD-1 treatments. ICI therapy boosts the body's natural defense against tumor by promoting the T cell specific immune response, and although it shows a lower toxicity respect to standard chemotherapy, it can lead to previously described AEs. AEs are in fact a consequence of an altered immunologic tolerance due to immune checkpoint disruption. The misdirected stimulation of the immune system toward a normal tissue due to a prolonged immune activation can lead to autoimmune-like/inflammatory side-effects. Delayed autoimmune toxicity can even emerge over time after discontinuing anti-PD-1 antibody treatment. Thus, in light of rapid increase in the number of patients receiving anti-PD-1 agents, a longer term follow-up of patients treated with ICIs would be recommended. To this aim, it would be suitable to comprise the period after cessation of therapy.

It has been recently demonstrated that pre-existing active rheumatic diseases heightened in patients receiving anti-PD-1 treatments (38). However, PD-1 inhibitors were responsible for lower toxicity compared to other immunotherapies, such as IL-2 and CTLA-4 blockade. In more detail, the majority of patients receiving monotherapy with PD-1 antagonists presented modest side effects respect to other immunotherapies, such as those involving IL-2. The severity, in particular of colitis, was lower for PD-1 antagonists respect to patients treated with anti–CTLA-4 mAb. Even though the combined treatment with anti-PD-1 plus anti–CTLA-4 mAbs resulted in increased response rates in patients less responsive to monotherapy, it resulted in a higher severity of side effects (39).

The analysis recently conducted by Le Burel et al. (40) through the investigation of the Registre des Effets Indésirables Sévères des Anticorps Monoclonaux Immunomodulateurs en Cancérologie (REISAMIC) registry, reported for the first time the onset of connective tissue diseases (CTD) in 4 out of a total of 448 patients treated with anti-PD-1/anti-PD-L1 agents. More specifically, two cases of Sjögren's syndrome and one case of cryoglobulinemic vasculitis as a complication of suspected Sjögren's syndrome, and one case of myositis positive for antinuclear antibodies^+^ (ANA^+^) were observed. Three of the patients were females and all had metastatic cancer. Two subjects had received anti-PD-1 agents and two anti-PD-L1 agents and no apparent symptom of CTD was detected before the treatment with ICIs. The correlation between anti-PD-1/PD-L1 cancer immunotherapy and CTD onset revealed the necessity to identify asymptomatic patients at risk of IrAEs (40). The participation of subjects presenting autoimmune disorders has been mostly excluded by immunotherapy clinical trials.

In this Review, we discuss specifically the role of PD-1/PD-L1 on Tregs in view of future therapeutic perspectives targeting this specific immunotype. Although these molecules are at high expression, the involvement of the pathway in the expansion and function of this cell population remains indeed to be fully elucidated (41).

## Tregs and PD-1 expression

The group of Sakaguchi et al. (42) discovered Tregs in 1995. Since their discovery, our knowledge regarding this population has widely increased. They represent a developmentally distinct subset of suppressor CD4^+^ T cells critically involved in the quality and magnitude of immune responses, in the establishment of peripheral tolerance through the inhibition of Teff cells and the suppression of the immune-mediated tissue destruction toward self-antigens. Treg activity occurs primarily at the site of inflammation where they are attracted by inflammatory signals. Several suppressor mechanisms are used by Tregs (Figure 1) (43, 44) depending on the physiological and inflammatory underlining condition (45, 46).

Tregs are able to modulate the immune response in an antigen-dependent and independent manner (47). The suppression of CD4^+^ T cell activities by Tregs is mediated by inhibitory cytokines including transforming growth factor β (TGF-β) and IL-10, the latter being important for its immunosuppressive activity at environmental interfaces (48). The release of IL-10 from Th1 cells is triggered by TGF-β1 (49), which inhibits the further cytokine synthesis and directly reduces the activity of Teffs (50). In addition, Cottrez et al. (49) found that IL-10 potentiates the response of activated T cells to TGF-β1 by modulating TGF receptor expression. TGF-β and IL-10 exert limited effect on Teff expansion.

This local qualitative cytokine composition in the inflammatory microenvironment is able to modulate the magnitude of the immune response that halts antigen presenting cell (APC) functions. In addition, the inhibitory activity of Tregs can occur also by cell–cell contact with pathogenic immunotypes at the sites of inflammation through CTLA-4, lymphocyte-activation gene 3 (LAG-3) (51) and PD-1 (52). CTLA-4, LAG-3, PD-1 as well as PD-L1 are indeed highly expressed on Tregs (44).

Tregs can be distinguished into two subpopulations: naturally occurring Tregs (nTregs) and adaptive or induced Tregs (iTregs). The development of nTregs occurs in the thymus, and in basal conditions they are mitotically quiescent (53). They necessitate antigenic stimulation to expand *in vivo* (53), but they do not need TCR engagement to execute their inhibitory tasks (54). Conversely, iTregs develop from CD4^+^ forkhead box protein 3 (Foxp3)^−^ naive T cells in the periphery following antigenic stimulation. Chen et al. (55) demonstrated the generation of iTregs from peripheral CD4^+^CD25^−^ naive T cells through TGF-β induction of transcription factor *Foxp3*. Foxp3 belongs to the forkhead/winged-helix transcription factor family and plays a key role in Treg cell development and immunosuppressive activity. Mice presenting a genetic defect in Foxp3 are characterized by dysfunctional Tregs and develop systemic autoimmune features resembling lupus-like disease.

The inhibition of CTLA4 signaling using anti-CTLA4 blocking antibody considerably altered Treg frequency leading to an increase in this cell population as demonstrated by Tang et al. (56) and highlighting a new role for CTLA4 in the modulation of Treg turnover.

In addition, nTregs show elevated levels of CD25, the expression of Foxp3 (57) and a TCR repertoire recognizing self-antigens.

Treg development in the thymus is fundamental for the stable *Foxp3* expression, which represents the principal transcription factor involved in the regulation and maintenance of Treg phenotype and function. Tregs in the thymus can indeed recognize self-antigens (57, 58). Treg population represents a heterogeneous cell population which complicates Treg isolation based on the markers CD4/CD25/Foxp3. Indeed, different microRNAs, transcription factors, chemokine receptors, cytokines, inhibitor molecules, and other immune-related proteins can be expressed on different Treg subpopulations depending on the pathological and environmental situation. Recently, different subpopulations within the Treg population have been recognized through the identification of many novel additional markers (59), such as CD45RA which allows to distinguish CD45RA^+^Foxp3^lo^ resting Tregs (rTregs), CD45RA^−^Foxp3^hi^ activated Tregs (aTregs), and cytokine-secreting CD45RA-Foxp3^lo^ non-suppressive Tregs (60). In addition to Tregs, other regulatory CD4^+^ T cells are present, such as Type 1 regulatory T cells (Tr1) and Th3 cells, characterized by suppressive activities but do not express Foxp3 [*rev. in* (61)].

In addition, the critical role played by Tregs during pregnancy has also been demonstrated (62). In more detail, during normal pregnancy circulating maternal Tregs specific for fetal antigens increase their number already in the early stage of pregnancy allowing the maintenance of tolerance toward foreign paternal alloantigens by the maternal immune system (63). Treg number is maintained high also after delivery, even though their reduction post-partum has been reported by several studies. Moreover, their quick proliferation during the subsequent pregnancies has been reported. Accordingly, a defective number as well as activity of Tregs have been often correlated with unexplained infertility, miscarriage and pre-eclampsia (64–67). A recent study performed by Care et al. (68) also revealed that a reduced Treg number was responsible for uterine artery dysfunction in mice.

Mutations affecting *Foxp3* have been identified in immune dysregulation, polyendocrinopathy, enteropathy X-linked syndrome (IPEX) syndrome characterized by non-functional Tregs (69). Similarly, Foxp3^−^ mutant scurfy mice and Foxp3^−^ null mice show the deficiency of CD4^+^CD25^+^ Tregs causing an aggressive lymphoproliferative autoimmune disorder which can disappear with Treg subset restoration. The addition of *Foxp3* transgene can also promote Treg differentiation in immunodeficient mice (56).

However, *Foxp3* expression is not specific to Tregs, but it has been described also on Teff lymphocytes. A reduction in Treg numbers or a defective function of this subpopulation causes the onset of autoimmune conditions in adult mice (46). Accordingly, several conditions in animal models including NOD and inflammatory bowel disease (IBD) mouse models can be reduced upon adoptive transfer of Tregs.

It has been observed that Treg generation as well as suppressive Treg properties and also Treg/Teff-cell interaction could be modulated at least in part by PD-1 expression (33) and by PD-1/PD-L1 binding.

In the presence of TGF-β, *Foxp3* expression is induced on naive CD4^+^ T cells generating iTregs (55, 70–72) which showed high levels of CD25, CTLA-4, and glucocorticoid-induced TNF receptor (GITR). Activated Tregs show PD-1 expression that has been identified on conventional T cells, even if at a lower level (73). The absence of PD-1 expression promoted autoimmune disorders in animal models and humans (4, 5, 74). PD-1 signaling in CD4^+^ Tregs is fundamental for the restriction of the number as well as for the suppression of Ag-reactive activity of Teff cells that accumulate in the periphery in response to an immunogenic stimulus (19).

Accordingly, the progression of many autoimmune disorders, including experimental autoimmune encephalomyelitis (EAE) (75), diabetes, and colitis, was promoted when the interaction between PD-1 and B7-H1 was inhibited (76, 77). Bedke et al. (52) demonstrated a significant increase of immunosuppressive activity of CD4^+^CD25^+^Foxp3^+^ Tregs upon EC contact mediated by PD-1 up-regulation on Tregs occurring during the extravasation of these cells from the blood into the inflamed tissue. The change of Treg phenotype was associated also with elevated IL-10 and TGF-β synthesis (52). Furthermore, recent evidences have highlighted the correlation between an altered function of Tregs and the development of an autoimmune condition as well as with skin tumors.

## The role of PD-1 in treg development and activity

Tregs are characterized by the expression of both PD-1 and PD-L1, which exert a role in the regulation of T cell tolerance (78). Even though PD-1/PD-L1 signaling pathway has been identified on Foxp3^+^ Tregs, its role in the regulation of their function and activity has not been fully elucidated (30).

Treg development from naive T cells could be promoted by the interaction occurring between DCs expressing PD-L1 and T lymphocytes (3). In addition, PD-1/PD-L1 binding reduced the generation of T cells, the release of cytokines and survival. Moreover, PD-1 prompts Foxp3 expression and enhanced Treg suppressive activity (79). However, the host environment and PD-1 signaling play a significant role in Treg development as demonstrated by the fact that APCs deficient for PD-L1 caused a diminished generation of Tregs from CD4^+^ T lymphocytes.

Raimondi et al. (73) observed that the regulated compartmentalization of PD-1 discriminates CD4^+^CD25^+^ resting Tregs from activated T cells. PD-1 signaling pathway is also important for the maintenance of the suppressive capacity of Tregs. Francisco et al. (79) reported indeed that iTreg cell differentiation, maintenance and function was induced by PD-L1 by sustaining and increasing the expression of Foxp3 in iTregs. PD-L1 promotes iTreg conversion through the inhibition of phosphatidylinositol-3-kinase/mammalian target of rapamycin (Akt/mTOR) signaling cascade and the simultaneous phosphatase and tensin homolog (PTEN) up-regulation. Accordingly, the development of iTregs from naive CD4^+^ T cells was critically lowered in PD-L1^−/−^ antigen-presenting cells, whereas a significant diminishment in iTreg development associated with a lethal immune-mediated pulmonary damage, was observed *in vivo* in PD-L1^−/−^PD-L2^−/−^ Rag^−/−^ recipients of naïve CD4 T cells. The involvement of PD-1 in Treg conversion was further confirmed by naive CD4 T cell transfer to Rag^−/−^ recipients receiving an anti–PD-L1 blocking antibody. The analysis of Treg cell development and immunopathology revealed an important defect in *de novo* iTreg differentiation in Rag^−/−^ mice receiving the anti–PD-L1 mAb treatment in respect to the control group. Moreover, a moderate lung inflammatory phenotype characterized the lungs of Rag^−/−^ mice administered with anti–PD-L1 mAb. Consistently with the results from PD-L1^−/−^PD-L2^−/−^ Rag^−/−^ recipients receiving naive CD4 T cells, defective iTreg differentiation as well as pulmonary inflammation develops in wild type Rag^−/−^ mice treated with anti–PD-L1 mAb. *Foxp3* expression as well as the suppressive Treg function were increased upon activation of T lymphocytes in presence of PD-L1-Ig (79).

In this regard, Amarnath et al. (80) reported the conversion of human Th1 cells into Tregs through the involvement of the PD-L1-PD-1 axis. In detail, conventional T cells or irradiated K562 myeloid tumor cells, characterized by a hyper-expression of PD-L1, were able to induce the conversion of TBET^+^ Th1 cells into Foxp3^+^ Tregs *in vivo*, preventing human-into-mouse xenogeneic GvHD (xGvHD) onset. Th1 cells could mediate lethal xGVHD when PD-1 expression on Th1 cells was halted or PD-1 signaling was inhibited. Hence, targeting PD-1 signaling through blockade of PD-1 expression or pharmacological inhibition of PD-1 signaling pathway could represent a potential strategy to increase T cell immunity against infection and cancer (80).

In order to investigate how Tregs inhibit antibody production, Gotot et al. (81) used transgenic mice expressing model antigens in the kidney demonstrating that the establishment of peripheral B-cell tolerance toward glomerular autoantibodies involved PD-1. More specifically, B cell suppression by Tregs do not need intermediate Th cells. In fact, the inhibition of autoreactive B cells by Tregs involved directly the interaction of both PD-1 ligands on Tregs with PD-1 on autoreactive B lymphocytes. The engagement of PD-1 suppressed both the activation and proliferation of self-reactive B cells, and promoted their apoptosis.

The study conducted by Wong et al. (82) investigated whether PD-1 expression could affect the generation of CD4^+^ Tregs by treating mice with a neutralizing antibody. The anti-PD-1 treatment diminished PD-1 expression on CD4^+^ Treg (PD1^lo^CD4^+^Treg) *in vivo*; the suppressive activity of CD4^+^ Tregs was indeed affected by PD-1 level. More specifically, the blockade of PD-1 induced the formation of adaptive regulatory CD4^+^CD25^+^ T cells. In fact, PD1^low^CD4^+^ Tregs showed a higher capacity to elicit B cell apoptosis and inhibit CD4^+^ helper T cells (T_h_) in respect to CD4^+^ Tregs presenting an elevated PD-1 expression (PD1^hi^CD4^+^Tregs) (82).

## Evidences of PD-1 and treg involvement in autoimmunity

An increasing number of studies support the involvement of PD-1 and Treg interactions in the onset of different autoimmune conditions such as insulin-dependent diabetes mellitus (Type 1 diabetes, T1D), psoriasis, vitiligo, systemic lupus erythematosus (SLE) and inflammatory bowel diseases (IBD). Here we summarize recent supporting literature on these conditions.

### Type 1 diabetes

Type 1 diabetes represents a multifactorial autoimmune disorder characterized by the destruction of pancreatic β cells by autoreactive T lymphocytes (83). Both genetics (84, 85) and environmental factors (86) are involved in T1D pathogenesis. The autoimmune response directed against pancreatic islet cells leads to a slow progressive and selective destruction of these cells (a condition identified as primary autoimmune insulitis) and, over the years, to a clinically manifested disease (87). In NOD mice, PD-1 blockade (88) or deficiency promoted both T1D development and induction of autoreactive T lymphocyte proliferation and their pancreatic infiltration (6). Recent studies have highlighted the correlation between the pharmacological treatment with PD-1 or PDL-1 antibodies (nivolumab or pembrolizumab) and T1D onset (89, 90). Mellati et al. (90) suggested that anti–PD-1, and possibly anti–PDL-1 antibody treatment could be responsible for a quick progression of autoimmune diabetes in human subjects characterized by an elevated underlying genetic predisposition to T1D, similarly to that observed in rodent models.

In T1D patients, Tregs showed a significant increase whereas Teffs were significantly diminished respect to controls. The observation that Treg/Teff ratio was higher in patients than in controls allowed to hypothesize that Tregs were functional in T1D patients (91). Concerning PD-1 expression on Tregs, no difference was found between patients and controls. Upon stimulation with CD3/CD28, Treg proliferation was defective in T1D subjects. In addition, healthy controls showed also a higher Teff proliferation. Concerning the ratio between Treg and Teffs, after 6 days from stimulation, the control group showed a significant increase respect to T1D group suggesting the reduced inhibitory functionality of Tregs in T1D patients respect to healthy subjects. Moreover, in T1D patients these cells presented reduced percentages of total PD-1^+^, PD-1^low^, and PD-1^high^ suggesting that reduced PD-1 expression and hence a defective PD-1/PD-L1 signaling pathway could lead to a deficient Treg activation (91).

A recent investigation conducted by Iijima et al. (92) studied for the first time the expression of PD-1 in circulating CD4^+^ and CD8^+^ T cells from fulminant T1D onset to 12 weeks after initiation of treatment. A consistent reduction was observed in circulating CD4^+^PD-1^+^ and CD8^+^PD-1^+^ T cells at the onset of fulminant T1D in two subjects with diabetic ketoacidosis (DKA) caused by T1D. Their number was restored upon treatment, as opposite to the number of CD4^+^CD25^+^FoxP3^+^ Tregs.

### Psoriasis

Psoriasis constitutes a chronic inflammatory skin disease mediated by multiple molecules and cells belonging both to the innate and adaptive immune arms. Psoriasis is characterized by a defective basal keratinocyte differentiation responsible for an enhanced proliferation and incomplete differentiation of these cells. One of the hallmark histologic features of psoriasis is the presence of neutrophils infiltrating the epidermis, where they are attracted by several chemotactic factors. The altered immune response characterizing psoriasis is due to a pathogenic cross-talk involving keratinocytes, DCs, and T lymphocytes. T cells have a key role in the initiation phase of the disease, especially those residing in the skin as tissue-resident memory T (TRM) cells. The inflammatory process is sustained by IL-17, IL-22, and TNF. Although the etiology of the disease remains to be fully elucidated, a combination of environmental and genetic factors could be responsible for an abnormal immune response. The identification of T cell subsets that are specifically involved in the pathogenic process has not been clarified yet (93). Myeloid-derived suppressor cells (MDSCs) are among the defective cell components with immunosuppressive activity believed to play a role in non-malignant inflammatory diseases, such as asthma, IBD, arthritis, and psoriasis (94). Soler and McCormick (94) demonstrated that PD-1 surface expression was reduced on monocytic MDSCs (Mo-MDSCs) from psoriatic patients; in addition, although the generation of Tregs from naive Teffs was induced both by psoriatic and control Mo-MDSCs, Tregs induced by psoriatic Mo-MDSCs showed a reduced suppressive activity. It is possible that T cell proliferation and hyper-activation is not limited due to alterations in psoriatic Mo-MDSCs (94).

Since keratinocytes express PD-L1 and PD-L2, Kim et al. (95) investigated whether their expression in terms of mRNA and protein levels on keratinocytes obtained through skin biopsies from psoriasis, allergic contact dermatitis (ACD), pityriasis rosea (PR), and lichen planus (LP) were altered respect to normal epidermis. Concerning psoriatic epidermis, PD-L1 and PD-L2 mRNA levels were consistently reduced respect to healthy epidermis. In psoriasis, PD-L1 protein expression was reduced compared ACD, PR, LP and normal epidermis. Psoriatic and normal epidermis showed no expression and minimal expression of PD-L2; conversely, it was enhanced in the other inflammatory skin disorders ACD, PR, and LP. These changes in psoriatic epidermis allow to hypothesize that PD-L1 and PD-L2 could contribute to the chronic dysregulated inflammatory process underlying psoriasis by promoting the continuous T cell activation. The normal expressions of PD-L1 could inhibit the hyper-activated state of T lymphocytes (95). Although merely few data are currently available concerning Tregs in psoriasis, Fujimura et al. (96) reported a reduction in PD-L1 expression on APCs in case of Treg depletion (96). The altered Treg function may lead to reduced PD-L1 and PD-L2 expression.

### Vitiligo

Vitiligo is a skin autoimmune disorder characterized by the destruction of melanocytes by antigen-specific T cells, causing a white patchy depigmentation. Vitiligo affects 0.5–2% of the population worldwide (97). Even though the etiology remains to be elucidated, the involvement of both genetic and environmental factors was hypothesized (98).

Miao et al. (99) demonstrated that the treatment of adult pre-melanosomal protein-1 (Pmel-1) vitiligo mice with a PD-L1 fusion protein reversed consistently the progression of depigmentation (99). This occurred through the activation and enhancement of Tregs in the skin. The increase of this cell population was observed also in the spleen and in the circulation. Treatment targeting PD-L1 blocked the immune process and was able to revert the depigmentation. PD-L1 fusion protein exerted even a more prolonged activity (until 8 weeks after the final treatment) in respect to CCL22 DNA and simvastatin treatments (2 weeks and 1 week, respectively). The immune response involving melanocyte-reactive T cells in vitiligo was inhibited *in vivo* upon PD-L1 protein therapy. By enhancing remarkably Treg abundance in the skin, this treatment was able also to revert depigmentation development in Pmel-1 vitiligo mice. Hence, PD-L1 fusion protein could represent a novel potential therapeutic strategy for patients with vitiligo (99).

### Systemic lupus erythematosus

SLE represents a severe systemic autoimmune disease characterized by the production of pathogenic autoantibodies directed against several self-antigens. It is responsible for the tissue inflammation and causes damage in several organs, such as the skin and kidneys (100).

The investigation conducted by Mesquita et al. (101) reported several alterations in the expression of critical surface molecules on Treg and Teff cells in 26 SLE patients with active disease compared to 31 with inactive disease and 26 healthy controls, despite healthy controls and SLE patients showed equivalent Treg cell frequency. More specifically, a higher CD40L^+^ Treg cell frequency, associated with reduced CTLA-4^+^ Treg and CD28^+^ Treg cell frequencies, characterized SLE subjects. However, a further characterization of Tregs based on the expression of regulatory, effector and activation molecules, including PD-1 expression, revealed no difference in terms of PD-1^+^ Treg cells as well as Treg/Teff ratio between SLE patients and healthy controls. Conversely, a reduced frequency of CTLA-4^+^ and CD28^+^ Tregs, together with a higher frequency of CD40L^+^ Tregs and an increased ratio of Treg/Teff CD40L^+^ cells, was observed in SLE patients. The frequency of CD40L^+^ Tregs was positively correlated with the SLE disease activity index. These alterations could play an important role in SLE pathogenesis (101).

### Inflammatory bowel diseases

IBD includes Crohn's disease (CD) and ulcerative colitis (UC), which are chronic inflammatory disorders affecting the gastrointestinal tract. Even though the exact pathogenesis remains to be elucidated, a defective regulation of the host immune response to intestinal flora in genetically susceptible individuals could be involved (102). Studies conducted both in mouse models and human patients support the involvement of Tregs in the IBD etiopathogenesis.

Recently, Alfen et al. (103) for the first time characterized *ex vivo* human intestinal type 1 regulatory T (T_R_1) cells. In particular, they observed that human intestinal Treg1 either expressing interferon (IFN)-γ and IL-10, revealed also the presence of C-C chemokine receptor type 5 (CCR5) and PD-1. These cells showed IFN-γ expression and were able to efficiently reduce T-cell proliferation and colitis symptoms. Conversely, intestinal IFN- γ –producing type 1 Tregs that co-expressed CCR5 and PD-1, were obtained from the inflamed guts of IBD patients and mice and showed the downregulation in the synthesis of the anti-inflammatory IL-10 cytokine. The diminished production of IL-10 by T_R_1 cells in response to the pro-inflammatory cytokines (IL-1β and IL-23) critically involved in colitis onset, was observed in both UC and CD patients and represent a common characteristic of IBDs. The abnormal intestinal inflammation characterizing IBD patients could be caused by the selective inhibition of IL-10 production by IL-10 and IFN-γ co-expressing IFN-γ^+^ T_R_1 cells in response to pro-inflammatory cytokines (103).

## Evidences for PD-1 pathway and treg involvement in cancer

A common feature of several different tumors is the ability to evade the host immune response (104). This phenomenon occurs through two well-recognized mechanisms: the negative modulation of CD4^+^ and CD8^+^ T cells by Foxp3-dependent Tregs and the expression of PD-L1 that can inhibit the antitumor activity of PD-1 positive CD8^+^ T lymphocytes (Figure 1).

Indeed the PD-1/PD-L1 interaction in the tumor microenvironment promotes tumor escape from immune surveillance and favors its growth (Figure 1). The anticancer immune response of T lymphocytes is inhibited by PD-L1 overexpression, a phenomenon observed in different tumor types i.e., breast adenocarcinoma, colon adenocarcinoma, and squamous cell carcinoma (105). Tumorigenesis and invasiveness are also enhanced *in vivo* following PD-L1 transgenic expression. Moreover, cancers showing a higher PD-L1 expression are even more resistant to specific CD8^+^ T cell–mediated lysis *in vitro*. In addition, an altered expression of PD-1 on CD4^+^ and CD8^+^ T cells could be responsible for Teff cell exhaustion in the tumor site (106–108). Specifically, PD-L1 could be involved in two mechanisms of evasion in cancer cells: innate and adaptive resistance. The over-expression of PD-L1, promoted by oncogenic and constitutively activated signals, including epidermal growth factor receptor (EGFR), protein kinase B (AKT) and Kirsten rat sarcoma viral oncogene homolog (KRAS) pathways, is responsible for an innate (tumor cell intrinsic) resistance (109–113). PDL-1 can be induced not only in cancer but also in immune cells (myeloid suppressor cells, dendritic cell, macrophage, and lymphocytes) in the tumor microenvironment by inflammatory signals, a mechanism identified as adaptive resistance (114, 115).

Park et al. (116) confirmed PD-1 up-regulation in tumor-infiltrating Teffs and Tregs compared to those at distant site from the tumor. In addition, these authors also reported the over-expression of multiple suppressive receptors, including T cell immunoglobulin mucin 3 (TIM-3), CTLA-4, GITR, and LAG-3, consistently with previous published data; indeed the higher expression of these receptors was reported in infiltrating lymphocytes in an immunosuppressive environment (117–122). These data allow hypothesizing a possible correlation between the up-regulation of these inhibitory receptors and the higher suppressive activity of tumor-infiltrating Tregs (116). Therefore, the host immune response is not able to eliminate cancer cells, which can proliferate and develop metastasis (116). In order to clarify the putative role of PD-1 in melanoma growth, Kleffel et al. (123) produced stable *Pdcd1* knockdown (KD) and *Pdcd1*-overexpressing (OE) B16 melanoma lines observing that melanoma-specific Pdcd1-KD and Pdcd1-OE showed a reduction and an increase in melanoma growth, respectively, in immunocompetent C57BL/6 mice respect to controls. Tumorigenesis was increased also upon binding of PD-1 by PD-L1. Accordingly, the inhibition of PD-L1 by using RNA interference (RNAi), blocking antibodies, or introducing two single point mutations in the two PD-1 signaling motifs ITIM and the immunoreceptor tyrosine-based switch motif (ITSM) located within the cytoplasmic region of PD-1, abolished the tumor growth in immunocompetent, immunocompromised and PD-1-deficient tumor graft recipient mice. These results support the crucial involvement of PD-1 in the efficient melanoma growth (123).

PD-1-PD-L1 blocking agents can restore tumor immunity targeting the immune alterations evoked by tumor in its microenvironment. In particular, PD-1-PD-L1 inhibitors enhanced the cytolytic activity of tumor-specific T cells, reduced suppressive cytokine IL-10 production, whereas enhanced pro-inflammatory cytokine synthesis, promoted the presence of Teffs and diminished the numbers and suppressive function of Tregs in the tumor site (35–38). Tregs have also been demonstrated to exert a cancer immunosuppressive activity in several murine tumor models (124).

A higher activity of Tregs can cause immunosuppression and lead to Th1 cell number reduction, favoring the onset and the progression of skin cancers. Upon binding between PD-1 and PD-L1, Th1, and Tc1 cells are inhibited and the synthesis of their cytokines (IFN-γ and IL-2) is reduced while T cell migration, proliferation, as well as the secretion of suppressive (IL-10) or cytotoxic mediators are inhibited (125).

The unprecedented and durable response rates described recently in a remarkable percentage of cancer patients, including treatment-refractory patients with advanced cancers, have allowed since 2011, the approval by US Food and Drug Administration (FDA) of several inhibitors of PD-1/PD-L1 axis in many cancers (126). In patients, the therapeutic blockade of PD-L1 contrasts effectively different tumors, such as classic Hodgkin lymphoma (HL), melanoma (127), non–small cell lung cancer (NSCLC), small cell lung cancer (128), urothelial carcinoma, renal cell carcinoma (RCC) (129), gastric carcinomas, and hepatocellular carcinoma (130). The first clinical trial investigating the effects of PD-1 blockade revealed a positive correlation between PD-L1 expression on tumor cells and therapeutic response (131–133).

The inhibition of Treg activity by blocking CTLA4 (134) and PD-1 (132) represents an effective therapeutic target in some cases of melanoma, especially if both proteins are targeted simultaneously (135, 136), despite the risk of dose-dependent toxicity and autoimmune disorders.

For this reason, in human cancer immunotherapy, only partial blockage of CTLA-4 has been recommended. PD-1 targeting led to a mild autoimmune condition, which resulted more severe in the simultaneous block of CTLA4 and PD-1 pathways (5).

## Conclusions

The strictly regulated interaction between inhibitory and activating receptors and their ligands plays a fundamental role in the establishment and maintenance of immune system homeostasis (14). Defects affecting cells involved in immuneregulation such as Tregs or altered expression of molecules on their surface or in their regulatory pathways can lead to the development of pathological conditions including autoimmune disorders or promote cancer progression by favoring the evasion of tumor cells from the host immune response. At the same time, these molecules or subpopulations could represent potential therapeutic targets, as demonstrated by the efficacy of therapeutic blockade of PD-1/PD-L1 pathway used in the management of different cancers in humans. Further investigations are necessary in order to fully comprehend the complex mechanisms underlying the onset of these pathological conditions. This would improve the efficacy of tailored approaches in the personalized treatment of autoimmune and cancer conditions.

## Author contributions

EG conducted the literature research, wrote the article. AF supervised the conduction of literature search and wrote the article.

### Conflict of interest statement

The authors declare that the research was conducted in the absence of any commercial or financial relationships that could be construed as a potential conflict of interest.
